# Marked peripheral eosinophilia due to prolonged administration of posaconazole

**DOI:** 10.1099/jmmcr.0.005100

**Published:** 2017-06-30

**Authors:** Maher Alharbi, Rae-Kiran Jhinger, Terence Wuerz, Andrew Walkty

**Affiliations:** ^1^​ Section of Infectious Diseases, King Abdulaziz Medical City, National Guard Health Affairs, Jeddah, Saudi Arabia; ^2^​ Department of Medical Microbiology and Infectious Diseases, University of Manitoba, Winnipeg, MB, Canada; ^3^​ Department of Internal Medicine, University of Manitoba, Winnipeg, MB, Canada; ^4^​ Diagnostic Services Manitoba, Winnipeg, MB, Canada

**Keywords:** posaconazole, peritoneal dialysis, eosinophilia, mucormycosis

## Abstract

**Introduction.** Posaconazole is a triazole antifungal that is used in the treatment of a variety of fungal infections, as well as in the management of mucormycosis (on an off-label basis). Eosinophilia associated with exposure to azole antifungals has been described rarely in the literature.

**Case presentation.** A 31-year-old male on peritoneal dialysis (PD) for end-stage renal disease, secondary to diabetic nephropathy, presented to hospital with abdominal pain after a trip to St Lucia. He was taken to the operating room, where the PD catheter was removed and an abdominal-wall abscess was debrided. *R* *hizopus* species was recovered on culture of the abdominal-wall tissue, and the patient was started on amphotericin B deoxycholate. He was subsequently stepped down to posaconazole, for a planned treatment duration of 12 months. Approximately 43 days after the initiation of posaconazole, it was noted that his peripheral eosinophil count started to rise. No other cause for the eosinophilia was identified. Posaconazole was discontinued, and the patient’s eosinophil count began to drop 2 days later. The temporal association of eosinophilia following initiation of posaconazole and the subsequent improvement after drug discontinuation suggests a probable causal relationship.

**Conclusion.** At the time of writing, there have been only two other published cases of azole-associated peripheral eosinophilia. In reporting this case, we hope to increase health-care provider awareness of this rare adverse event. For patients receiving prolonged therapy with posaconazole, periodic monitoring of the complete blood count with differential may be considered.

## Abbreviation

PD, peritoneal dialysis.

## Introduction

Posaconazole is a triazole antifungal with *in vitro* activity against a broad range of fungi, including species belonging to the order *Mucorales* [1]. Posaconazole is indicated for the prophylaxis of invasive aspergillosis among high-risk patients, and the prophylaxis and treatment of infections caused by *Candida* spp. [2]. It has also been used off-label in the management of mucormycosis, generally as salvage therapy or as step-down treatment following a course of liposomal amphotericin B [3–5]. Eosinophilia related to therapy with the azole class of antifungals has been rarely described in the literature [6, 7]. Herein, we report a case of peripheral eosinophilia in a peritoneal dialysis (PD) patient who was receiving posaconazole for the treatment of a PD-cathter related soft tissue infection caused by *Rhizopus* species.

## Case Report

A 31-year-old male with type II diabetes mellitus complicated by diabetic nephropathy and end-stage renal disease was admitted to a tertiary care hospital (Winnipeg, MB, Canada) in December 2015 with malaise and abdominal pain, following a 9 day trip to St Lucia. The patient had been receiving PD since January of 2015 and while on vacation he used PD fluid purchased in the Caribbean. Physical examination at the time of admission revealed a 15×7 cm area of fluctuant induration surrounding the tunnelled catheter site, with associated purulent drainage. The patient was admitted to hospital and empiric antimicrobial therapy with vancomycin, piperacillin/tazobactam (Pip/Tazo 2.25 g IV q8hr) and tobramycin was initiated. A central line was placed for intermittent haemodialysis in anticipation of PD catheter removal.

The patient was taken to the operating room 1 day post-admission for removal of the PD catheter. Intra-operatively, a 10 by 20 cm area of necrotic infected tissue around the PD catheter site was debrided down to the abdominal-wall fascia. The catheter cuff and abdominal-wall tissue were submitted to the microbiology laboratory (Health Sciences Centre, Winnipeg, MB, Canada). On direct microscopic examination of the abdominal-wall tissue with a calcofluor stain, fungal elements were visualized. The microscopic features of the hyphae were consistent with the organism belonging to the order *Mucorales. Rhizopus* species was subsequently recovered on culture. No bacteria were seen on direct Gram stain, and culture of the tissue for aerobic and anaerobic bacteria did not yield any additional pathogens. At 3 days post-admission, the patient was started on amphotericin B deoxycholate via intravenous infusion and antibacterial therapy was discontinued. He was taken back to the operating room twice for further debridement of necrotic tissue and for control of bleeding. There was no evidence of intra-abdominal infection on abdominal computed tomography scans performed at the time of admission and 10 days post-admission.

**Fig. 1.**
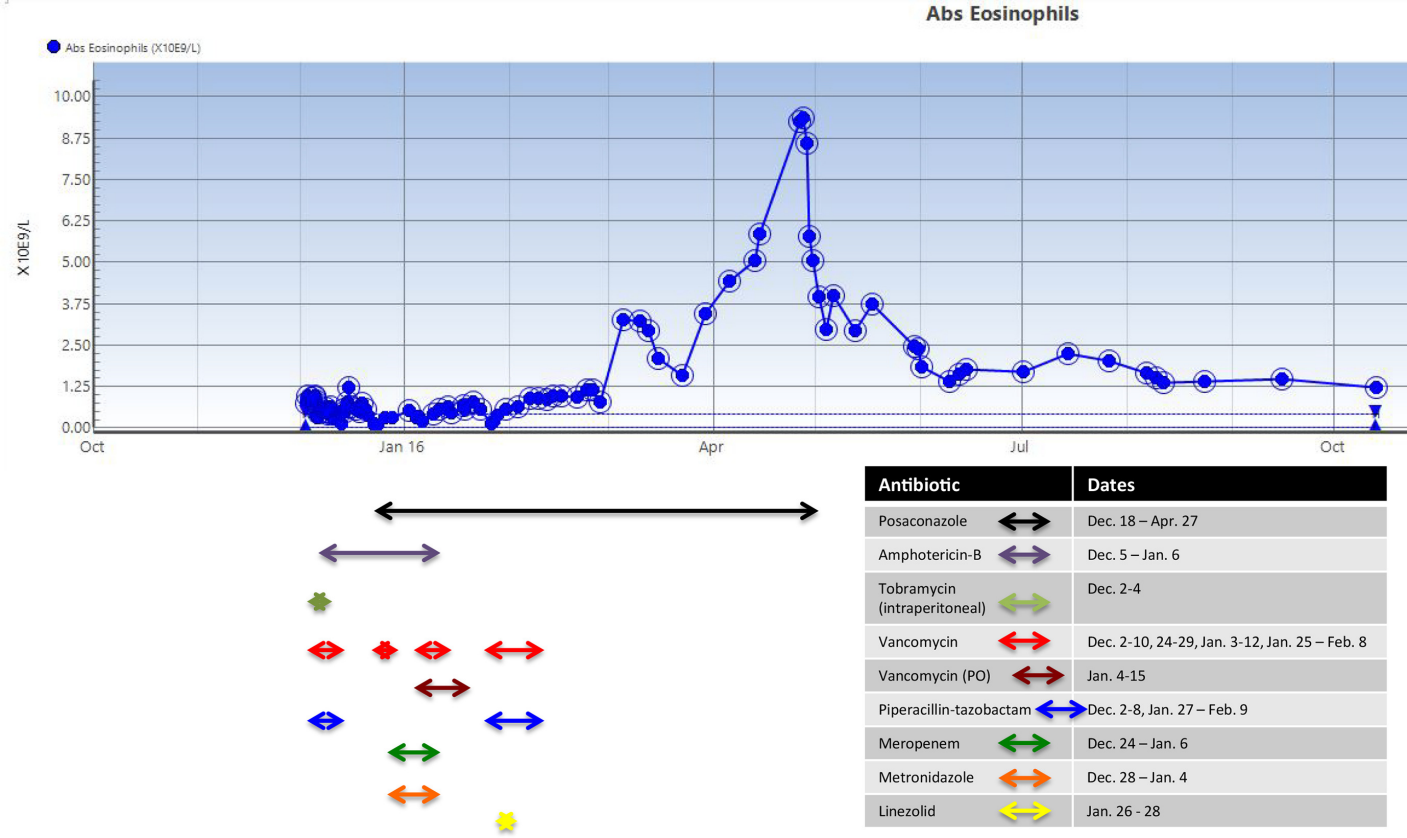
Absolute peripheral eosinophil count in relation to posaconazole and other antimicrobials.

The patient continued treatment with amphotericin B deoxycholate for a total of 33 days. He initially received a dose of 1 mg kg^−1^ daily via intermittent infusion, and after 2 days this was changed to 2 mg kg^−1^ daily via continuous infusion. On day 17 post-admission, intravenous posaconazole, 300 mg every 12 h, was added. Therapy with posaconazole overlapped with amphotericin B to allow posaconazole levels to reach steady state. The patient was stepped down to posaconazole oral suspension at 21 days post-admission at a dose of 400 mg twice daily. This was changed to posaconazole tablets at a dose of 300 mgorallytwice daily at 37 days post-admission, for a planned total duration of 12 months’ therapy in consultation with the infectious diseases service (Health Sciences Centre, Winnipeg, MB, Canada) (see Fig. 1 for the time course of the antimicrobial therapy). The tablet dosage was higher than that typically recommended in the literature [8], and was selected based on the severity of infection and a lack of published data on the optimal posaconazole dose for mucormycosis.

The patient’s hospital course was complicated by severe deconditioning, and an episode of health-care-associated pneumonia, which resolved after a course of piperacillin/tazobactam (2.25 g IV q8hr) and vancomycin. Beginning around day 59 post-admission, a slow rise in the patient’s absolute eosinophil count was noted, for which no obvious explanation was determined on history or physical examination. At the time of re-consultation with the infectious diseases service at 134 days post-admission, the peripheral absolute eosinophil count was 5.85×10^9^cells l^−1^ corresponding to a white blood cell count of 16.3 (35.9 % eosinophils). At its maximum, 147 days post-admission, the absolute eosinophil count was 9.35×10^9^ cells l^−1^ with a corresponding white blood cell count of 19.0 (49.3 % eosinophils). It was noted that the patient’s peripheral absolute eosinophil count began to rise approximately 43 days after the initiation of posaconazole. A medication review was performed, which did not reveal any other potential medications as a cause for the patient’s eosinophilia. Serology for *Strongyloides* infection, requested given the recent travel to St Lucia, was negative. The haematology service reviewed (Health Sciences Centre, Winnipeg, MB, Canada) the patient and did not find a primary haematological cause for the eosinophilia.

It was suspected that posaconazole was the cause of the eosinophilia and the drug was discontinued at 146 days post-admission. At this time, the patient had completed close to 5 months of antifungal therapy. A decision was made to withhold further antifungal treatment given the extensive debridement early in the admission, the prolonged course of antifungal therapy to that date and the current clinical status of the patient. After the posaconazole had been held for 2 days, the eosinophil count began to drop (Fig. 1). One month after discontinuation of posaconazole, the absolute eosinophil count had decreased to 2.46×10^9^ cells l^−1^. The patient was not re-challenged with posaconazole.

## Discussion

The order *Mucorales* contains several medically important genera, including *Rhizopus*, *Mucor*, *Cunninghamella*, *Apophysomyces*, *Lichtheimia* and *Rhizomucor* [9, 10]. Fungi belonging to the *Mucorales* are ubiquitous throughout the environment, but invasive infections caused by these organisms are rare [9]. The most common clinical manifestations of infection caused by the *Mucorales* include rhino-orbital-cerebral disease, pulmonary disease, cutaneous disease, gastrointestinal disease and disseminated disease [10]. Infections mainly occur among immunocompromised patients or those with underlying diabetes mellitus, and are associated with significant morbidity and mortality [3, 10]. In a review of 929 published cases of *Mucorales* infection between 1940 and 2003, diabetes mellitus was the most common underlying condition (36 %), followed by malignancy (17 %) [11]. Chronic renal insufficiency was present in 4 % of patients [11]. PD-related infections caused by the *Mucorales* have been infrequently described in the literature [10, 12]. Seventeen published cases of PD-related mucormycosis were reviewed by Rathai *et al.* in 2014 [12]. For the 12 cases where culture results were available, the fungi most frequently recovered were *Rhizopus* spp. (42 %, 5/12) and *Mucor* spp. (42 %, 5/12). Among cases with a clearly documented outcome, the crude mortality rate within 2 months of infection was 40 % (6/15) [12].

Amphotericin B is recommended as the antifungal of choice for patients with mucormycosis [3–5]. Liposomal amphotericin B has been favoured for the treatment of mucormycosis over amphotericin B deoxycholate in order to deliver a high dosage while limiting nephrotoxicity [3]; however, a 24 h infusion of amphotericin B deoxycholate may offer an alternative treatment option in accomplishing this goal [13]. Posaconazole may play a role in salvage therapy, or as a step-down option for patients treated with amphotericin B who are clinically improving [4, 5, 14]. Posaconazole is a newer triazole antifungal agent [1]. It is marketed as an intravenous formulation, a delayed release tablet and an oral suspension [2]. In general, posaconazole is associated with a favourable toxicity profile. The adverse effects most frequently reported include gastrointestinal complaints (nausea, vomiting, abdominal pain and diarrhoea) and headache [2]. In clinical trials, the frequency of haematological abnormalities (anaemia, neutropenia) among patients receiving posaconazole has been comparable to patients treated with other azoles (fluconazole, itraconazole) [2, 15–17]. Eosinophilia attributed to posaconazole exposure has not been documented among clinical trial participants [2, 15–17].

There are only two cases previously published in the literature of azole-associated peripheral eosinophilia [6, 7]. In 2004, Vishnubhotla *et al*. described a patient who developed fever and eosinophilia while receiving therapy with voriconazole for pulmonary aspergillosis [6]. The patient’s total leukocyte count peaked at 78×10^9^ cells l^−1^ with 10.6 % eosinophils at 11 days following initiation of voriconazole treatment. The leukocytosis and fever improved within 2 days of stopping the offending agent [6]. The patient was not re-challenged with voriconazole or another azole. Subsequently in 2014, Grzegorczyk *et al*. published a case of a patient with chronic pulmonary aspergillosis who presented with peripheral eosinophilia and eosinophilic colitis after more than 5 years of treatment with voriconazole [7]. The eosinophilia and associated gastrointestinal symptoms resolved after stopping voriconazole. The patient subsequently had a recurrence of the eosinophilia and diarrhoea after 15 months of posaconazole treatment [7]. Symptomatic improvement was again documented with discontinuation of azole therapy.

### Conclusion

In summary, the prolonged use of posaconazole appears to have been associated with the development of eosinophilia in this case. Based on the Naranjo scale [18], the eosinophilia in the patient described here would be classified as a probable adverse reaction to posaconazole. The marked eosinophilia arose after an extended period of treatment with posaconazole, it improved with discontinuation of the drug, no alternative cause was identified that could have explained the eosinophilia, and there are other published case reports of eosinophilia occurring secondary to azole therapy. Limitations in drawing a causal relationship here include the higher-than-standard dosing of posaconazole, the extended-release tablet that was used and the lack of a drug re-challenge. While eosinophilia related to azole therapy appears to be an uncommon event, it is important that clinicians be aware that it may occur. We suggest that for those patients receiving therapy with posaconazole, periodic monitoring of the complete blood count and differential may be considered in order to identify this complication and intervene, if necessary.

## References

[1] Torres HA, Hachem RY, Chemaly RF, Kontoyiannis DP, Raad II (2005). Posaconazole: a broad-spectrum triazole antifungal. Lancet Infect Dis.

[2] Merck (2014). Noxafil Prescribing Information.

[3] Cornely OA, Arikan-Akdagli S, Dannaoui E, Groll AH, Lagrou K., European Society of Clinical Microbiology and Infectious Diseases Fungal Infection Study Group, European Confederation of Medical Mycology (2014). ESCMID and ECMM joint clinical guidelines for the diagnosis and management of mucormycosis 2013. Clin Microbiol Infect.

[4] Riley TT, Muzny CA, Swiatlo E, Legendre DP (2016). Breaking the mold: a review of mucormycosis and current pharmacological treatment options. Ann Pharmacother.

[5] Tacke D, Koehler P, Markiefka B, Cornely OA (2014). Our 2014 approach to mucormycosis. Mycoses.

[6] Vishnubhotla P, Ibrahim RB, Abidi MH, Chandrasekar PH (2004). Fever and eosinophilia associated with voriconazole. Ann Pharmacother.

[7] Grzegorczyk B, Murata Y (2014). Peripheral eosinophilia and eosinophilic colitis during long‐term azole therapy for pulmonary aspergillosis. JMM Case Rep.

[8] Mckeage K (2015). Posaconazole: a review of the gastro-resistant tablet and intravenous solution in invasive fungal infections. Drugs.

[9] Garcia-Hermoso D, Alanio A, Lortholary O, Dromer F, Landry ML, Jorgensen JH, Pfaller MA, Carroll KC, Funke G (2015). Agents of systemic and subcutaneous mucormycosis and entomophthoromycosis. Manual of Clinical Microbiology.

[10] Farmakiotis D, Kontoyiannis DP (2016). Mucormycoses. Infect Dis Clin North Am.

[11] Roden MM, Zaoutis TE, Buchanan WL, Knudsen TA, Sarkisova TA (2005). Epidemiology and outcome of zygomycosis: a review of 929 reported cases. Clin Infect Dis.

[12] Rathi M, Sengupta U, Yadav TD, Kumar S (2014). Zygomycetes peritonitis in ambulatory peritoneal dialysis: case report and review of the literature. Indian J Nephrol.

[13] Falagas ME, Karageorgopoulos DE, Tansarli GS (2013). Continuous versus conventional infusion of amphotericin B deoxycholate: a meta-analysis. PLoS One.

[14] Vehreschild JJ, Birtel A, Vehreschild MJ, Liss B, Farowski F (2013). Mucormycosis treated with posaconazole: review of 96 case reports. Crit Rev Microbiol.

[15] Cornely OA, Maertens J, Winston DJ, Perfect J, Ullmann AJ (2007). Posaconazole vs. fluconazole or itraconazole prophylaxis in patients with neutropenia. N Engl J Med.

[16] Ullmann AJ, Lipton JH, Vesole DH, Chandrasekar P, Langston A (2007). Posaconazole or fluconazole for prophylaxis in severe graft-versus-host disease. N Engl J Med.

[17] Vazquez JA, Skiest DJ, Nieto L, Northland R, Sanne I (2006). A multicenter randomized trial evaluating posaconazole versus fluconazole for the treatment of oropharyngeal candidiasis in subjects with HIV/AIDS. Clin Infect Dis.

[18] Naranjo CA, Busto U, Sellers EM, Sandor P, Ruiz I (1981). A method for estimating the probability of adverse drug reactions. Clin Pharmacol Ther.

